# The Grapevine *VvPMEI1* Gene Encodes a Novel Functional Pectin Methylesterase Inhibitor Associated to Grape Berry Development

**DOI:** 10.1371/journal.pone.0133810

**Published:** 2015-07-23

**Authors:** Vincenzo Lionetti, Alessandro Raiola, Benedetta Mattei, Daniela Bellincampi

**Affiliations:** 1 Dipartimento di Biologia e Biotecnologie “C. Darwin”, Sapienza Università di Roma, Rome, Italy; 2 Dipartimento Territorio e Sistemi Agroforestali, Università di Padova, Legnaro (PD), Italy; University of Minho, PORTUGAL

## Abstract

Pectin is secreted in a highly methylesterified form and partially de-methylesterified in the cell wall by pectin methylesterases (PMEs). PME activity is expressed during plant growth, development and stress responses. PME activity is controlled at the post-transcriptional level by proteins named PME inhibitors (PMEIs). We have identified, expressed and characterized VvPMEI1, a functional PME inhibitor of *Vitis vinifera*. VvPMEI1 typically affects the activity of plant PMEs and is inactive against microbial PMEs. The kinetics of PMEI-PME interaction, studied by surface plasmon resonance, indicates that the inhibitor strongly interacts with PME at apoplastic pH while the stability of the complex is reduced by increasing the pH. The analysis of *VvPMEI1* expression in different grapevine tissues and during grape fruit development suggests that this inhibitor controls PME activity mainly during the earlier phase of berry development. A proteomic analysis performed at this stage indicates a PME isoform as possible target of VvPMEI1.

## Introduction

Pectins are structurally complex polysaccharides that account for nearly 35% of the primary cell wall of dicots [1]. Homogalacturonan (HG), one of the main component of pectins, is a polymer of α-1.4-linked galacturonic acid residues. HG is secreted into the cell wall as a highly methyl esterified polysaccharide and is de-methylesterified by apoplastic pectin methyl esterases (PMEs) [2–4]. PMEs are encoded by the large protein family PF01095 (http://pfam.xfam.org/family/PF01095), belonging to CAZy class 8 of carbohydrate esterases (CE8, www.cazy.org). The activity of PMEs can produces long stretches of acidic HG which, through Ca^++^-mediated crosslinks, form rigid “egg-box” structures that stiffen the cell wall [5]. The pectin methylesterification is critical for tissue integrity, wall plasticity and cell adhesion [6–10] and biotic and abiotic stress responses [3,11,12]. PME activity is efficiently regulated by endogenous pectin methylesterase inhibitors (PMEIs) which belong to the large multigene protein family PF04043 (http://pfam.xfam.org/family/PF04043) that includes the invertase inhibitors (INHs). PMEIs and INHs share several structural properties, such as the conserved position of four cysteine residues engaged in disulfide bridges and a very similar up-and-down four-helical bundle fold, although their target enzymes are not related [13]. Inhibition of PME by PMEI occurs trough the formation of an inactive high affinity and reversible stoichiometric 1:1 complex [13]. PMEIs are active against PMEs of plant origin and are ineffective against microbial enzymes. High resolution three-dimensional structure of the PME-PMEI complex revealed that most of the residues important for the interaction with the inhibitor are conserved in plant PMEs but not in fungal and bacterial enzymes, thus providing an explanation for the lack of inhibition of PMEI against microbial PMEs [13].

PMEI was identified for the first time in kiwi fruits [14] and later in several other plants like Arabidopsis, pepper, broccoli, banana, wheat and tomato [2,15–21]. Recent evidence demonstrates the role of PMEIs in a number of growth and developmental processes including apical meristems development [22], cell and organ size [10,23], growth acceleration [24] and fruit development and ripening [18,20,25]. In particular, the PMEIs from *Solanum lycopersicum* (SolyPMEI) and from *Actinidia deliciousa* (AdPMEI) have been proposed to control methylesterification of pectin during fruit development as well as fruit softening during ripening [26–29]. Plants may temporally express PMEIs to modulate the pectin structure and degradation during infection [3]. It has been shown that the overexpression of PMEIs in Arabidopsis, wheat and tobacco results in a lower level of PME activity, a higher degree of pectin esterification and a concomitant reduced susceptibility to fungal, bacterial and viral pathogens [4,23,30,31]. A higher methylesterification makes pectin less susceptible to hydrolysis by microbial cell wall degrading enzymes and, as a consequence, microbial growth is reduced. PMEIs also have potential application in agriculture and food technology [25,32,33]. Plant pectin methylesterase activity increases during different stages of wine and marc production leading to the release of high levels of methanol in the final products [34–36]. PMEI was proposed as a tool to reduce methanol formation in grape must and marc and in products derived by fermentation and distillation [37].

By using functional genomics and biochemistry approaches, we here demonstrate that the genomic sequence XP_002272964.1 from grape (*Vitis vinifera*) encodes a functional PMEI, VvPMEI1, closely related to the kiwi fruit inhibitor AdPMEI [38]. The VvPMEI1 expressed in *Pichia pastoris* typically affects the activity of plant PMEs and is inactive against microbial PMEs. The kinetic parameters of PMEI-PME interaction, determined by surface plasmon resonance, indicate that the inhibitor strongly interacts with PME at apoplastic pH while the stability of the complex is reduced by increasing the pH. The analysis of *VvPMEI1* gene expression in different grapevine tissues suggests that this inhibitor controls PME activity in flowers and at early phases of grape berry development. By proteomic analysis we identified a PME isoform, mainly expressed in the early phase of berry development, possibly participating with VvPMEI1 in the modulation of pectin methylesterification at this specific stage.

## Materials and Methods

### Gene identification and cloning

Putative pectin methylesterase inhibitors of *Vitis vinifera* were searched and identified in NCBI database (http://www.ncbi.nlm.nih.gov/protein). The amino acid sequences were aligned using ClustalW software (http://www.ebi.ac.uk/Tools/msa/clustalw2/). The molecular weight and isoelectric point of VvPMEI1 were calculated by Compute pI/Mw (http://web.expasy.org/compute_pi/). The signal peptide cleavage site was predicted by SignalP program (http://www.cbs.dfu.dk/service/SignalP). Cellular localization was predicted using MultiLoc program (http://abi.inf.uni-tuebingen.de/Services/MultiLoc/). Glycosylation prediction was performed by NetNGlyc 1.0 (http://www.cbs.dtu.dk/services/NetNGlyc/)

### Gene Expression Analysis

Grape tissues (*Vitis vinifera* cv. Trebbiano) were immediately frozen in liquid nitrogen and stored at -80°C until the analysis. Total RNA was extracted from 400 mg of different tissues according to the method described by Reid KE et al [39] using a CTAB-Spermidine extraction buffer. Total RNA (2μg) was DNase-treated by adding 2 μl of RQ1 DNase Reaction buffer 10X (Promega), 2 μl of RQ1 RNase-free DNase 1 u/μl (Promega), nuclease-free water to a final volume of 18μl and incubated 30 min at 37°C. The reaction was stopped with 1μl of RQ1 DNase Stop Solution (Promega) at 65°C for 10 min. For cDNA synthesis, DNase-treated RNA (1μg) was reverse-transcribed using the Improm-II Reverse Transcription System according to Promega manufacturer’s instructions.

Real-time quantitative PCR analysis was performed using an iCycler Real-Time PCR detection system (CFX96 Bio-Rad) according to the manufacturer’s guide. qRT-PCR analysis was performed by using a CFX96 Real-Time System (Bio-Rad). Complementary DNA (corresponding to 50 ng of total RNA) was amplified in a 30-μL reaction mix containing 1× GoTaq Real-Time PCR System (Promega) and 0.4 μm of each primer. Three technical replicates were performed for each sample, and data analysis was done using LinRegPCR software. Expression levels of *VvPMEI1*, relative to Elongation Factor 1, were determined using the Pfaffl method [40]. The synthesized *cDNA* were amplified using the following oligonucleotide primers:

*VvPMEI1* forward (5’- GTCCTGGCATCCACATATCC -3’) and

*VvPMEI1* reverse (5’- CAGTGCGAGGATCAGACTT -3’);

VIT_07s0005g00730 forward (5’- TGGACCACTTTCCAATCGGG -3’) and

VIT_07s0005g00730 reverse (5’- AAGCGGCACAGGTAGAAGAC -3’)

*EF1-α* forward (5’- ACCACTGGTGGTTTTGAAGC -3’) and

*EF1-α* reverse (5’- TGTTGTGGCATCCATCTTGT -3’).

### VvPMEI1 expression in *P*. *pastoris* and purification

Genomic DNA was isolated from grape leaves (100mg) using the NucleoSpin Plant Kit from Macherey-Nagel, Germany. The region of *VvPMEI1* encoding the predicted protein was amplified by PCR from genomic DNA (50ng) using *Pfu* DNA polymerase (Promega) with the following primer pairs: *VvPMEI1* forward (5’-AACAATGAGTTGACTGAGATTTG-3’) and *VvPMEI1* reverse (5’-TTATCCTATCAAACGCTTAGAG-3’). The amplification product was purified with Wizard SV Gel and PCR Clean-Up System (Promega) and cloned into the pGEM-T easy vector (Promega) according to manufacturer’s instructions and then sequenced. The region of *VvPMEI1* encoding the predicted mature protein was amplified by PCR using the recombinant plasmid pGEM-T:VvPMEI1 as template with forward (5’-ATGCGAATTCAACAATGAGTTGACTGAGATTTG-3’) and reverse (5’ATGCGGTACC TTATCCTATCAAACGCTTAGAG-3’) primers. The PCR product was cloned between the EcoRI and Kpn1 sites (underlined in the above primer sequences) into the pPICZαA vector and the resulting vector used to transform *P*. *pastoris* strain X-33 by electroporation using the BTX ECM 630 electroporator (Harvard Apparatus Company). Transformed *P*. *pastoris* cells, grown to saturation in BMGY medium (Invitrogen) at 28°C with shaking at 250 rpm, were harvested by centrifugation, suspended in BMMY medium (Invitrogen) and grown for 72 hrs. Methanol was added every 24hrs to a final concentration of 0.5% (v/v). Finally, cells were pelleted by centrifugation at 10000*xg* for 15 min and the supernatant assayed for PME inhibitory activity.

To purify the inhibitor, supernatant from the transformed *P*. *pastoris* cultures was precipitated in ammonium sulfate as previously described [18]. The precipitated protein were dialyzed against 20 mM Tris-Cl, pH 7.9 and loaded onto a ResourceQ 1ml column (Pharmacia Biotech) and fractions eluted with a linear gradient of NaCl from 0 to 0.5M at a flow rate of 1ml/min. The fractions containing VvPMEI1 inhibitory activity were collected, pooled, loaded onto a MonoQ column (HR 5/5, Pharmacia) and eluted with a linear gradient of NaCl, as described above, using an FPLC system (Pharmacia). The purity of the recombinant VvPMEI1 was checked by SDS-PAGE and silver staining and fractions containing VvPMEI1 were pooled and concentrated by ultrafiltration on Centricon 3 filters (Amicon). The recombinant VvPMEI1 was de-glycosylated using 0.2 mU of N-glycanase F (Genzyme, Boston, MA).

### Protein MS/MS analysis

MS/MS analysis of recombinant VvPMEI1 was performed on protein after SDS-PAGE separation.

The band excised from Coomassie stained gels was in-gel digested with trypsin (Promega) according to the procedure described by Shevchenko [41]. After enzymatic digestion, peptides were concentrated using ZipTip C18 reverse phase micro-columns (Millipore, Bedford, MA, USA) and analyzed by LC-MS/MS. The peptides were eluted over 180 min at 300 nl/min. using a 0–60% acetonitrile gradient in 0.1% formic acid using an Ultimate 3000 nano-chromatography pump (Thermo-Fisher Scientific). The peptides were eluted into a LTQ Orbitrap Discovery mass spectrometer (Thermo-Fisher, Bremen, Germany) operated in a data dependent mode. MS was acquired at 30.000 FWHM resolution in the FTMS (using a target value of 5 x10^5^ ions) and MS/MS was carried out in the linear ion trap. Five MS/MS scans were obtained per MS cycle. Spectra were analyzed using Sequest (Thermo Fisher Scientific, San Jose, CA, USA; version 1.3.0.339). Sequest was set up to search the *Vitis vinifera* proteome database (Uniprot.org) assuming trypsin as the enzyme with 2 missed cleavage allowed. Sequest was searched with a fragment ion mass tolerance of 0.60 Da and a parent ion tolerance of 10.0 ppm. Fixed modification of carbamidomethyl cysteine and variable modifications of oxidized methionine were considered in the search.

For the proteomic analysis tissues were ground using a mortar and pestle and incubated at 4°C for 3 hours in a buffer containing 20mM sodium acetate, 1M sodium chloride and 1:100 v/v protease inhibitor (P9599; Sigma), pH 5.5. After centrifugation at 15000*xg* for 15 min at 4°C the supernatant was collected and protein concentration determined. Protein extracts were digested in solution with trypsin (Promega); the peptides were concentrated using StageTip C18 reverse phase micro-columns (Millipore, Bedford, MA, USA) and analyzed by LC-MS/MS. Peptides were eluted over 180 min at 300 nl/min. using a 0–60% acetonitrile gradient in 0.1% formic acid. Mass spectra were analyzed using the MaxQuant Software package. Raw data files were searched against the NCBInr proteome database Viridiplantae (containing 1,878,311 sequence entries) assuming trypsin as the enzyme with 2 missed cleavage allowed. Maxquant was searched with a fragment ion mass tolerance of 0.60 Da and a parent ion tolerance of 10.0 ppm. Fixed modification of carbamidomethyl cysteine and variable modification of oxidized methionine were considered in the search. MaxQuant identifications required FDR<0.01. Proteins that contained similar peptides and could not be differentiated based on MS/MS analysis alone were grouped.

### VvPMEI1 inhibitory activity

Inhibitory activity of VvPMEI1 was assayed by agar diffusion assay as previously described [15,42]. In particular, 1% (w/v) agarose was added to (w/v) 0.1% Apple pectin (Sigma 76282; 70–75% esterification) and dissolved in 12.5 mM citric acid and 50mM Na_2_HPO_4_ buffer, pH 6.3. The solution (25 mL) was cast into 9.5 mm Petri dishes and allowed to gel at room temperature. Wells with a 4 mm diameter were obtained in the gel with a cork borer. Protein extracts (2 μg in 20 μL) or 50mU of purified PME1 from tomato, purified recombinant PME from *Aspergillus aculeatus* (a gift from K. Schnorr; Novo Nordisk A/S, Bagsværd, Denmark) or commercial orange peel PMEs (Sigma-Aldrich) were loaded in each well in the absence or presence of 20 ng of purified recombinant VvPMEI1. Plates were incubated at 30°C for 16 hrs and stained with 0.02% ruthenium red dye (Sigma) for 45 min. The plates were extensively de-stained by several washes with water and the diameter of the stained zones, resulting from the de-methylesterification of pectin in the gel were measured with IMAGE J software [43].

### Determination of PME activity

Different grapevine tissues were grinded using a mortar and pestle and incubated at 4°C for 3 hours in a buffer containing 20mM sodium acetate, 1M sodium chloride and 1:100 v/v protease inhibitor (P9599; Sigma), pH 5.5. After centrifugation at 15000*xg* for 15 min at 4°C the supernatant was collected and protein concentration determined. PME activity in different grapevine tissue was quantified by a modified alcohol oxidase/acetyl acetone procedure [44] adapted for a microplate assay. For each sample, 4 μg of proteins in 15 μl of 20mM sodium acetate buffer pH 6.0 were mixed to 50μl of 0.5% Apple pectin (Sigma 76282; 70–75% esterification) dissolved in the same buffer. To assay the inhibitory effect of VvPMEI1 against PME activity of grape berry at phase I of development different amounts of VvPMEI1 were pre-incubated for 15 min with proteins extracted from the unripe berry. The reaction was performed at 30°C for 3 hours and blocked by boiling at 100°C for 10 min. The solution was incubated in 96 micro wells plate and after addition of 35 μl of alcohol oxidase (0.03 units in assay buffer; Sigma), the samples were incubated at room temperature for 15 min on shaker. Thereafter, 100 μL of a mixture containing 0.02 M 2.4-pentanedione in 2 M ammonium acetate and 0.05 M acetic acid was added. After 10 min of incubation at 68°C, samples were cooled on ice and absorbance was measured at 412 nm in a microplate reader (Cary 50 MPR microplate reader, Varian). The methanol (MeOH) content was estimated as the amount of formaldehyde produced from methanol by alcohol oxidase and quantified by comparison with a MeOH standard calibration curve.

### SPR analysis

All SPR measurements were performed with the SensíQ Pioneer biosensor system at a controlled temperature of 25°C. Running buffer for experiments contained either 10 mM NaOAc (pH 5.5) or 10 mM HEPES (pH 7.5), 0.1 M NaCl, 0.005% (w/w) Tween-20. A COOH5 sensor chip (SensíQ Technologies) was used for the assays. The COOH5 sensor chip was installed and conditioned according to manufacturer's protocol. Tomato PME was immobilized using a standard amine coupling method. The method involved activation by injecting 20mM N-ethyl-N′-(3-dimethylaminopropyl) carbodiimide (EDC) with 5mM N-hydroxysuccinimide (NHS) followed by an injection of 25 μg/mL protein solution in 10mM sodium acetate buffer, pH 4.3. Activated surfaces were capped by injecting 1M ethanolamine pH 7.5 for 3 min. Reference channel (FC2) was left unmodified to serve as a reference for non-specific binding to the surface chemistry. Dilution series of VvPME1I was prepared in running buffer including concentrations: 300.0, 150.0, 75.0, 37.5, 18.7, 9.4, 4.7, and 2.3 nM and a buffer blank. Each sample was injected over the flow-cells for 5 min of contact time and the dissociation of the bound protein was observed under buffer flow for 5 min. Regeneration of the sensor surface was performed by injecting HEPES-buffered saline (HBS), pH 8.5 for 1 min after each VvPMEI1 injection. A second assay was performed using the Fast Step injection in dilution series in running buffer. SPR response curves from the FC2 were subtracted from the PME (FC3) channel curve. HBS blank curves were then averaged and subtracted from the VvPME1 curves. Dose–response plots were constructed as previously described [45]

## Results and Discussion

### Identification of *VvPMEI1*

**Fig 1:**
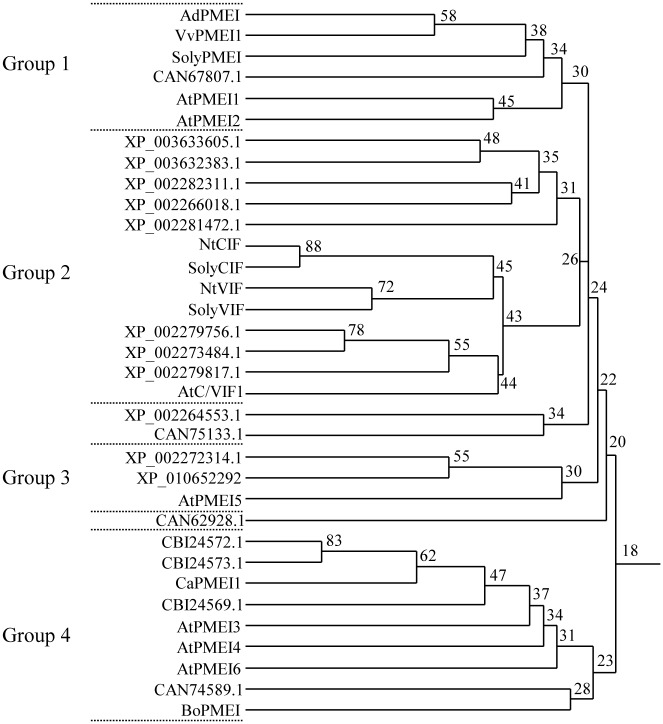
Homology tree of several *Vitis vinifera* protein sequences annotated as putative invertase/pectin methylesterase inhibitors. In the analysis the functionally characterized PMEIs and INHs are also included. Multiple sequence alignment was performed using DNAman software package (Lynnon Biosoft). Numbers at branch points represent % of identity.

A draft genome sequence of grapevine (*Vitis vinifera* L.) has been published for both a near-homozygous line [46] and a highly heterozygous clone [47] of the Pinot Noir variety. A search on NCBI database (http://www.ncbi.nlm.nih.gov/protein) was performed to identify genes encoding *Vitis vinifera* PMEIs. The analysis revealed a number of sequences predicted to belong to the plant invertase/pectin methylesterase inhibitor family (http://pfam.xfam.org/family/PF04043). A homology tree of the *V*. *vinifera* amino acid sequences was generated, including all the INHs and PMEIs so far functionally characterized in dicotyledonous plant. Four major protein groups were identified (Fig 1): the grape proteins included in Group 1 are XP_010660323, hereafter named VvPMEI1, and the VITISV_037797 (CAN67807.1). Interestingly, VvPMEI1 groups in a distinct cluster together with the best characterized PME inhibitor from kiwi (AdPMEI: P83326) [13,25,29,48]. The higher amino acid sequence identity (58%) indicates that these two genes are orthologues. In this group three biochemically characterized PMEIs are also included: SolyPMEI from *Solanum lycopersicum* [18] and two PMEI isoforms from *Arabidopsis thaliana*, AtPMEI1 (At3g17220) and AtPMEI2 (At1g48020) [15,49]. The evidence that AdPMEI and SolyPMEI play a role during fruit development and ripening [18,29] and that both Arabidopsis PMEIs are involved in pollen tube growth [50] suggests that VvPMEI1 could be involved in these physiological processes also in grapes. Group 2 includes a large number of *V*. *vinifera* proteins with sequences closely related to the already characterized functional cell wall/vacuolar inhibitor of fructosidases (C/VIF) suggesting their possible function as INHs [51]. Group 3 shows two *V*. *vinifera* deduced amino acid sequences (XP_002272314.1 and XP_010652292) related to the characterized AtPMEI5 (At2g31430) from *A*. *thaliana* involved in root emergence [52,53]. Group 4 identifies two closely related *V*. *vinifera* sequences, CB24572.1 and CBI24569.1, sharing higher level of identity with CaPMEI1 from pepper, previously reported to be involved in plant resistance to biotic and abiotic stresses [16]. The same group includes Arabidopsis AtPMEI3 (At5g20740) expressed in apical meristems and affecting primordia formation [22], AtPMEI4 (At4g25250) involved in the regulation of growth acceleration in dark-grown seedlings [24], AtPMEI6 (At2g47670) involved in seed maturation and germination [54] and the pollen specific BoPMEI from broccoli with a role in pollen tube growth [17]. The distribution of VvPMEIs in different groups indicates a large diversity of these proteins and suggests that PMEIs with distinct functional roles could be expressed in *V*. *vinifera*.

The nucleotide sequence of *VvPMEI1* gene of 546 bp was amplified by PCR using genomic DNA isolated from grape leaves and the amplicons were sequenced. The VvPMEI1 nucleotide sequence obtained (S1 Fig) reveals a 100% identity with the predicted mRNA sequence; XM_010662021.1 [46]. The gene is located in LOC100260329 locus in the fourteenth chromosome and lacks closely related paralogues in the grapevine genome [47]. The 5’ UTR nucleotide sequence of *VvPMEI1* gene was analyzed using PLACE database (http://www.dna.affrc.go.jp/PLACE/), in order to identify putative cis-acting DNA elements with regulative functions (S2 Fig). There are a number of CAAT and TATA responsive elements, feature of genes expressed during fruit development and ripening [18,55,56]. Hormone responsive elements, such as TAACGTA for gibberellin, CATATG for auxin, TATTAG for cytokinin and AWTTCAAA for ethylene, are also present [57–59]. All these elements are characteristic of the promoters of *SolyPMEI* and *AdPMEI* genes, which expressions are regulated in fruit during development and ripening [18,29]. In addition, the pollen-specific activation element AGAAA [60] is repeated different times in *VvPMEI1* promoters. This element is also highly present in the promoter sequences of *SolyPMEI* and in pollen-expressed *AtPMEI1*, *AtPMEI2* and *BoPMEI1* [17,18,49]. The *VvPMEI1* promoter also shows GAAAAA, GTTAGTT and TGTCA elements playing important roles in transcriptional regulation of defence genes expressed in response to fungal and bacterial pathogens [61–63].

### *VvPMEI1* gene encodes a *V*. *vinifera* pectin methylesterase inhibitor

**Fig 2:**
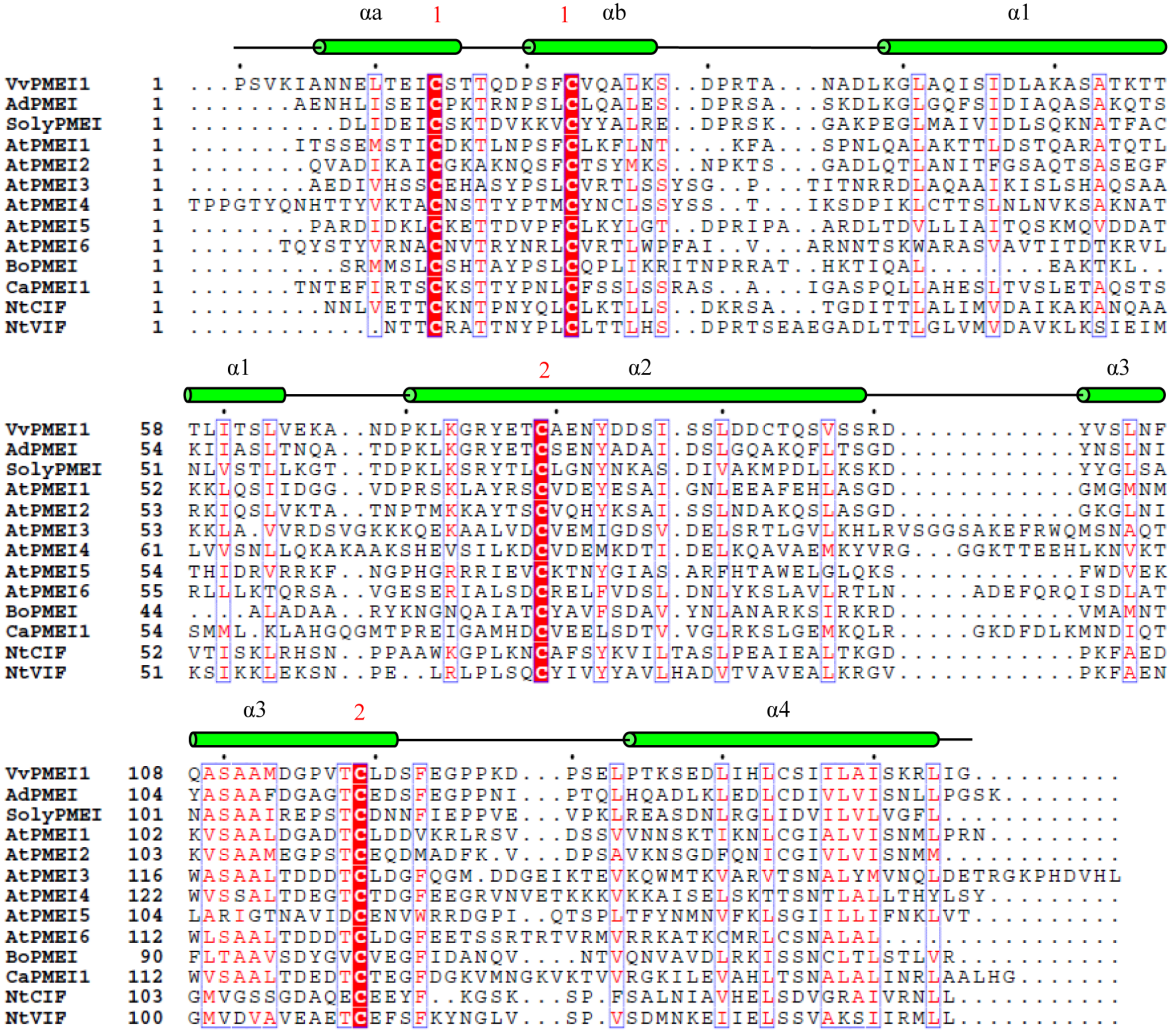
Alignment of amino acid sequences of functionally characterized PMEIs from different plant origins. The VvPMEI1 amino acid sequence was aligned with PMEIs from Arabidopsis (AtPMEI1: At1g48020, AtPMEI2: At3g17220, AtPMEI3: At5g20740, AtPMEI4: At4g25250, AtPMEI5: At2g31430 and AtPMEI6: At2g47670), kiwi (AdPMEI:P83326), pepper (CaPMEI1: ABG47806), tomato (SolyPMEI: SGN-U601352) and broccoli (BoPMEI:Q45TJ7). Tobacco cell wall and vacuolar invertase inhibitors (NtCIF: CAA73333 and NtVIF: CAA73334) were also added for comparison. The alignment performed using ClustalW was manually adjusted according to PsiPred secondary structure predictions. Alignment was drawn by using the ESPript program. The secondary structure elements, as elucidated in AdPMEI crystal structure, are indicated at the top of the alignment. Invariant residues are red shadowed and similar residues are colored in red and boxed. Numbers 1 and 2 at the bottom denote disulfide bridges connecting the four conserved Cys residues.

*VvPMEI1* gene is predicted to encode a protein of 181 amino acid residues including a signal peptide domain of 25 amino acids whose putative cleavage site is located between Arg 25 and Pro 26 (S1 Fig). VvPMEI1 has a theoretical molecular mass of 16.7 kDa and has an acidic isoelectric point of pH 4.64. A prediction analysis indicated for VvPMEI1 an extracellular localization (http://abi.inf.uni-tuebingen.de/Services/MultiLoc2). The amino acidic sequence of VvPMEI1 was analyzed and aligned with those of functionally characterized PMEIs and of two tobacco invertase inhibitors (Fig 2). VvPMEI1 holds the four conserved cysteine residues typically engaged in the formation of two disulfide bridges, important to stabilize both the αa and αb helices of the hairpin loop and the α2 and α3 helices of the four helical bundle structure of PMEIs [13,64] (Fig 2). VvPMEI1 has also a conserved Thr-118 residue previously demonstrated to strengthen the AdPMEI-PME1 interaction at the acidic apoplastic pH [13], a typical SAA amino acid motif in α3 helix, and a C-terminal hydrophobic region of six amino acids involved in the stabilization of the four-helical bundle structure of the protein [13]. Like other PMEIs, VvPMEI1 lacks in α3 helix the PKF motif, which defines a sequence fingerprint critical for invertase-INH interaction, as well as lacks the contiguous Ala and Glu residues, highly conserved in invertase inhibitors and contributing to the enzyme-inhibitor complex formation [63].

**Fig 3:**
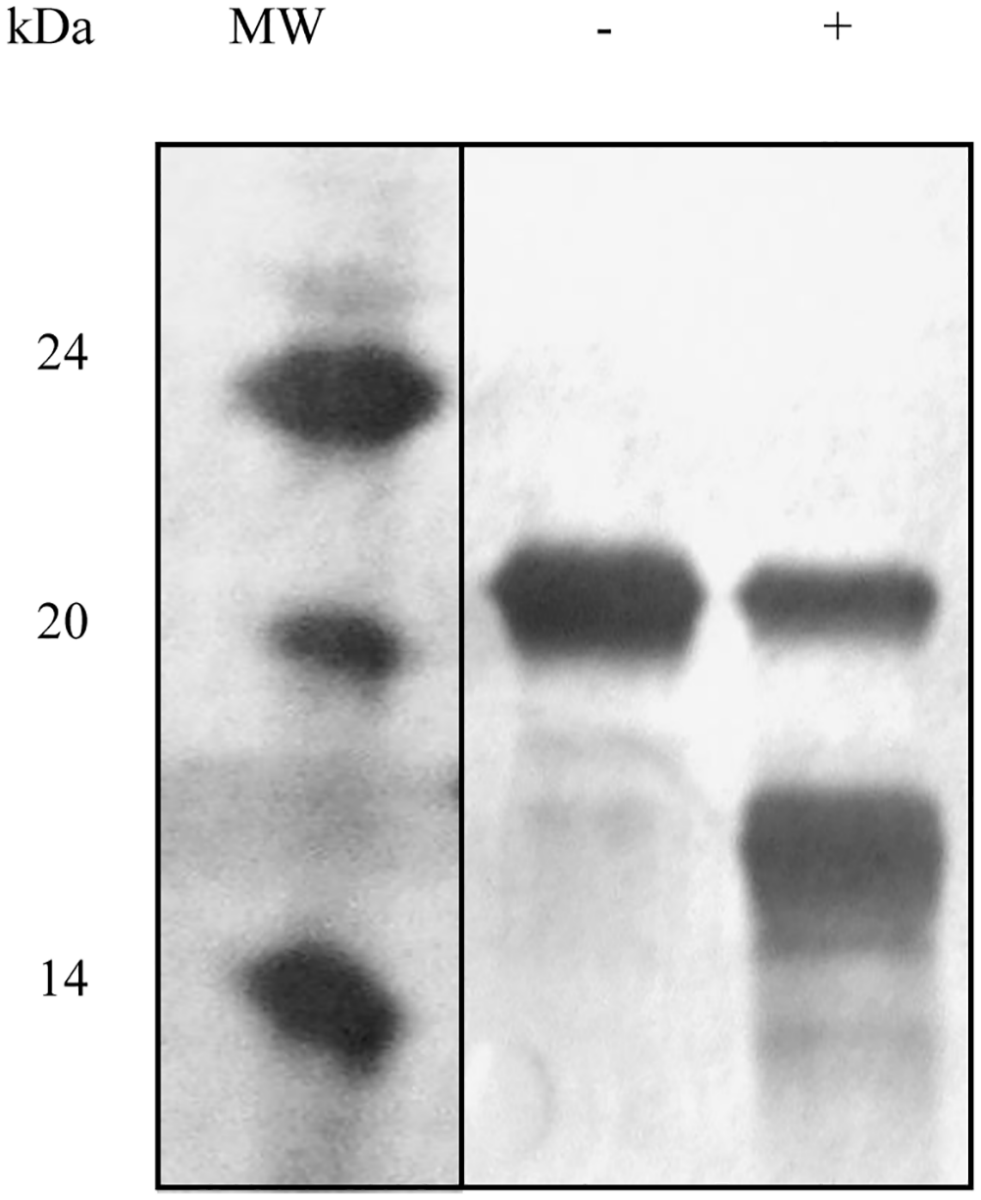
SDS-PAGE analysis of purified VvPMEI1. VvPMEI1 was loaded before (-) and after (+) endo N-glycosidase digestion: (Mw) molecular weight marker. The proteins were separated by SDS-PAGE and gel was stained with silver nitrate.

To biochemically characterize the inhibitor and to establish its specificity of recognition, the region of VvPMEI1 encoding the mature protein of 156 amino acids was cloned into the pPICZαA vector and expressed in *P*. *pastoris*. VvPMEI1 accumulated in the Pichia culture filtrate (30 mg/l) after methanol induction. VvPMEI1 was purified to homogeneity and showed a single band with an apparent molecular mass of 21 kDa by SDS-PAGE, higher than that predicted for the mature protein (Fig 3). The reduction of 4kDa of the molecular mass of the recombinant VvPMEI1 after treatment with endo-N-glycosidase F, indicated that recombinant inhibitor is glycosylated (Fig 3).

The amino acid sequence of the recombinant VvPMEI1 was determined by mass spectrometry (LC-MS/MS) after trypsin digestion. The identified fragments revealed an optimal matching with the deduced amino acid sequence and the coverage of 62% of the entire sequence of the protein (S3 Fig). The absence of two trypsin peptides in the result of the analysis suggests their glycosylation.

**Fig 4:**
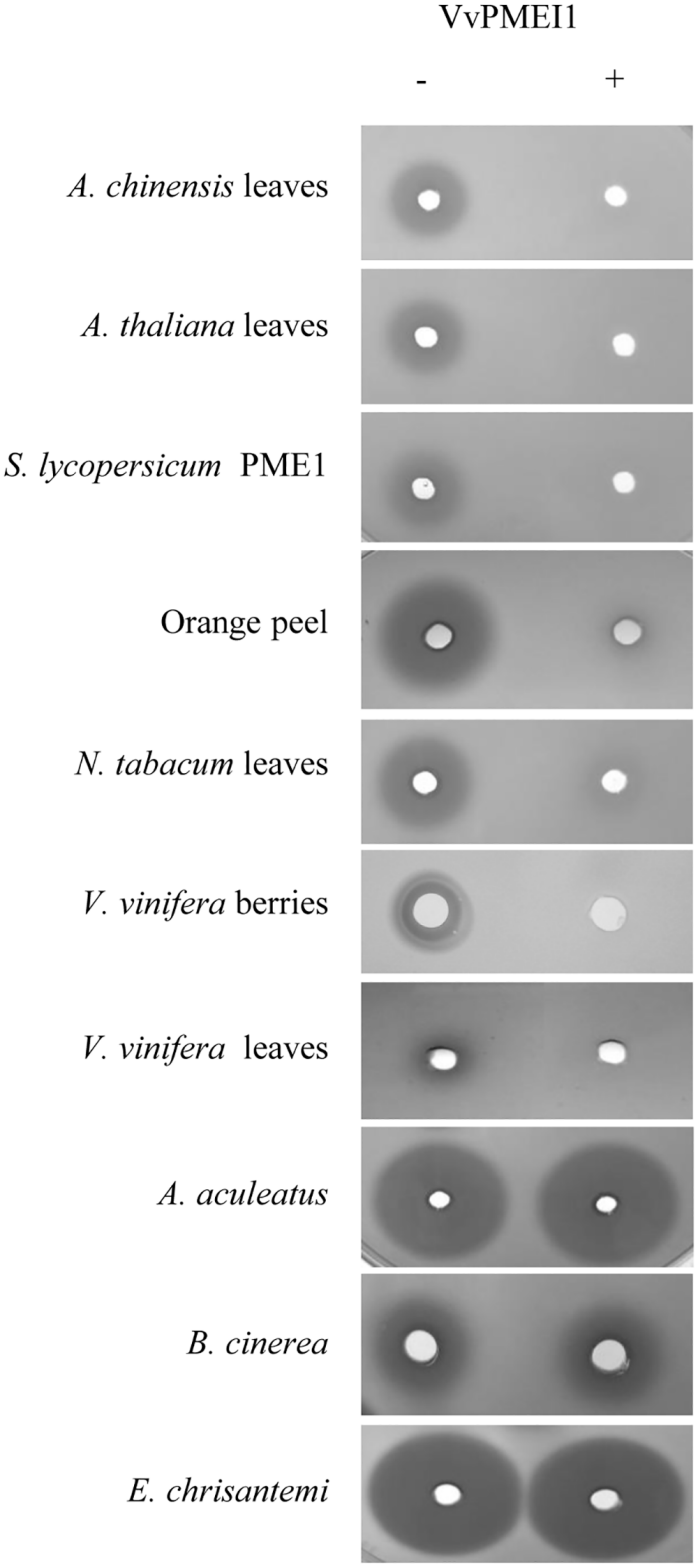
Target specificity of VvPMEI1. Gel diffusion assay showing PME activity from various origins in the presence (+) or absence (-) of VvPMEI1. The representative image of at least three independent experiments is shown.

Different PMEIs isoforms have been proposed as efficient tools for a number of food industry applications such as the stabilization of juice fruits and concentrates [25,32,65,66] or for reducing methanol formation in grape must and marc [37]. For this purposes, the inhibitory activity and target specificity of VvPMEI1 and its thermic and pH stability were assayed by gel diffusion assay [42]. PME activities from grape leaves and berries were inhibited by VvPMEI1 (Fig 4). The inhibitor was also effective against the tomato PME1 isoform (PME1; Swissprot accession number P14280), PME from orange peel and against PME activities extracted from kiwi, Arabidopsis and tobacco leaves. As other PMEIs so far characterized, VvPMEI1 was inactive against fungal and bacterial PMEs. VvPMEI1 maintains the inhibitory activity after treatment at 40°C, while loses 50% of its activity at 50 and 60°C and is inactivated at 70°C (Table 1). The inhibitory activity was stable at pHs of 5.5 and 6.5 and is lowered by 15% at pH 7.5 (Table 2). All these features make the protein suitable for several food industry applications.

**Table 1:** Inhibitory activity (%) of VvPMEI1 incubated at different temperatures for 10 min against tomato PME1.

	40°C	50°C	60°C	70°C
VvPMEI1	100	50.0±4.5	50.3±3.0	0

The inhibition observed at 40°C was taken as 100%. Each point represents the mean value ± SD of three repeated measurement.

**Table 2:** Inhibitory activity (%) of VvPMEI1 towards tomato PME1 as a function of pH.

	pH 5.5	pH 6.5	pH 7.5
VvPMEI1	100	100	84.0±2.6

The inhibition observed at pH 5.5 was taken as 100%. Each point represents the mean value ± SD of three repeated measurement.

### VvPMEI1-PME1 interaction analyses

**Fig 5:**
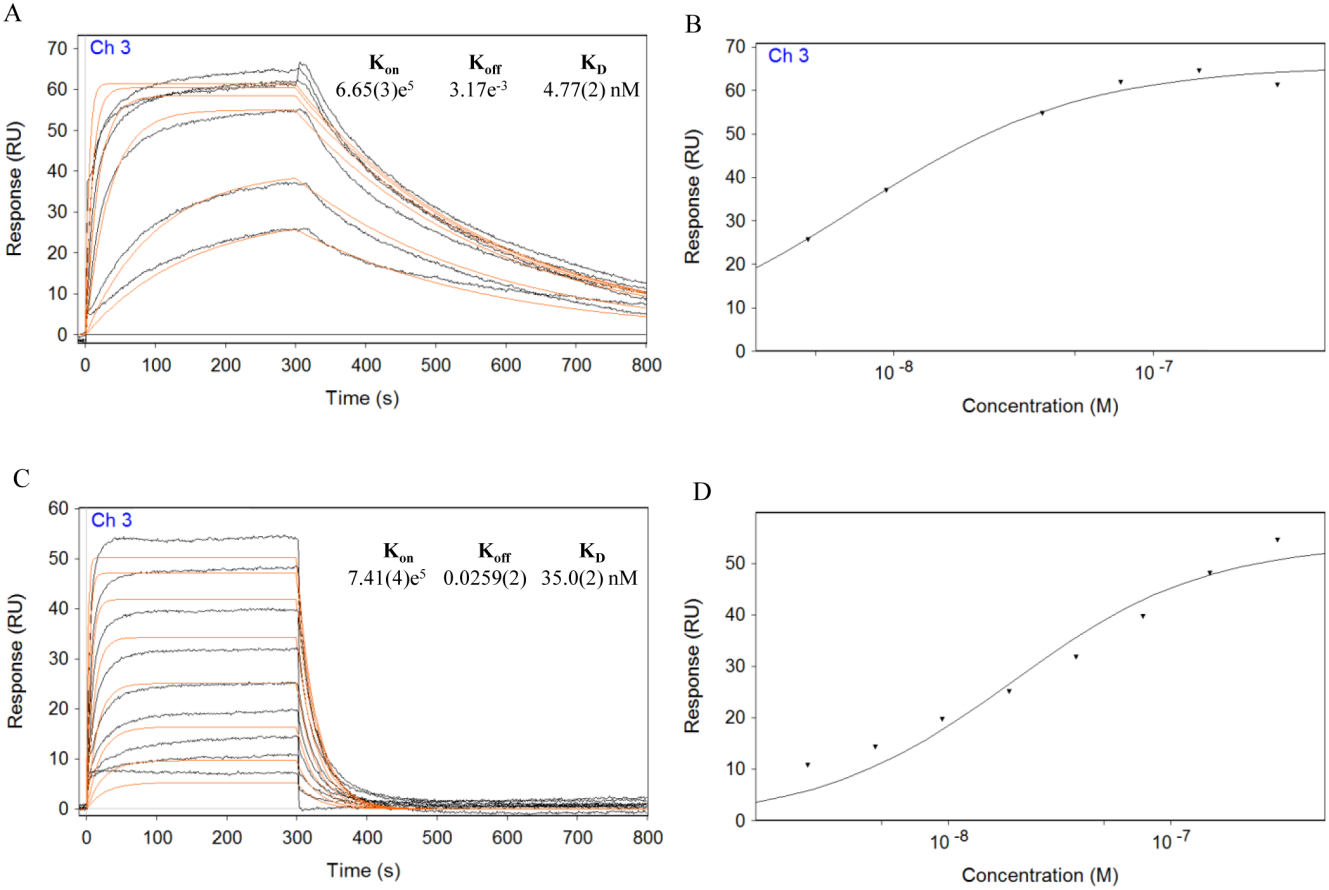
SPR analysis of VvPMEI1 and PME1 interaction. Different VvPMEI1 concentrations were injected over the surface (300.0, 150.0, 75.0, 37.5, 18.7, 9.4, 4.7, 2.3 nM). (A) and (C) show sensorgrams (black) obtained at pH 5.5 and 7.5 respectively. Data were fit to the simple 1:1 interaction model, and the resulting fit is shown in red. (B) and (D), concentration dependent binding curves for the interaction at pH 5.5 and 7.5 respectively.

**Fig 6:**
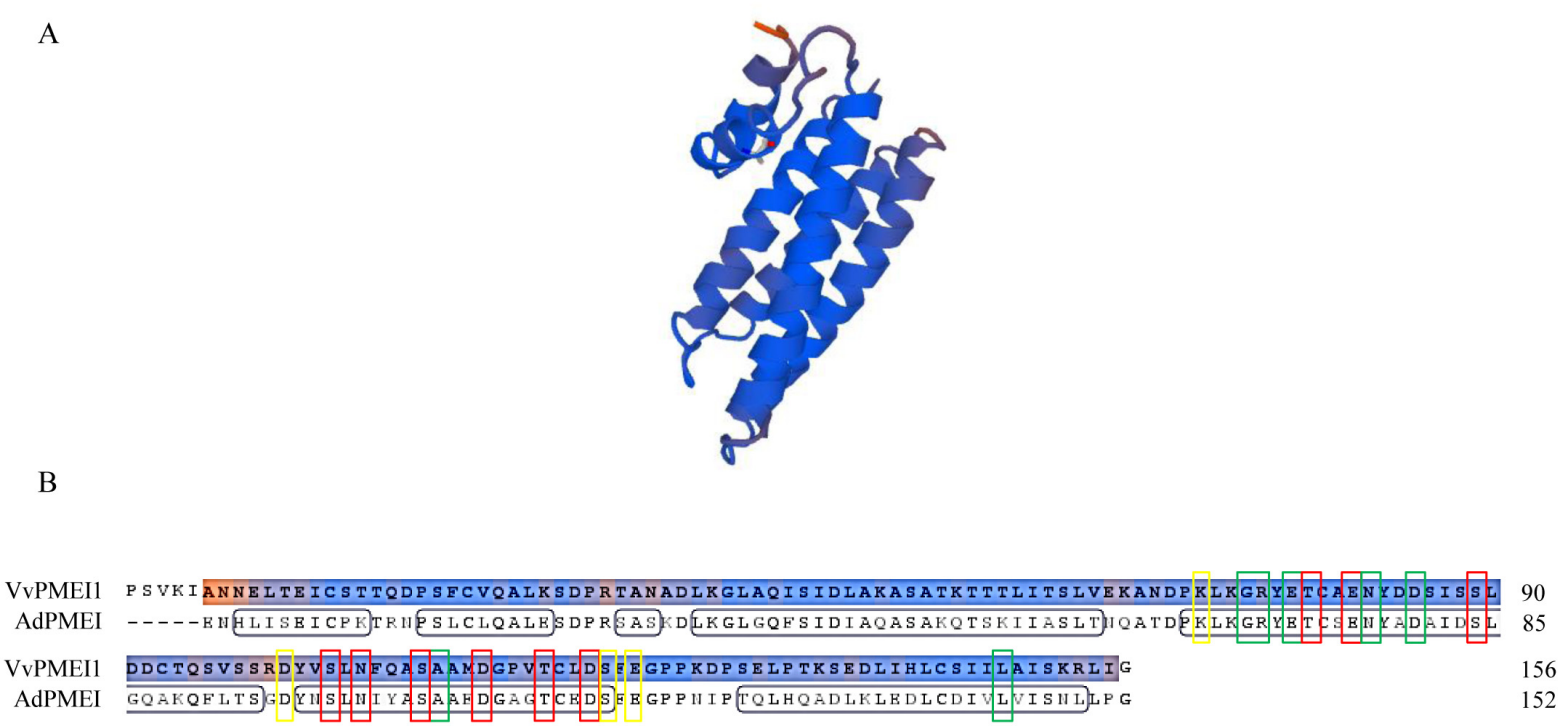
Homology model of VvPMEI1. The model of VvPMEI1 (top) was based on the AcPMEI template using the Swiss Model algorithm. Sequence comparison of VvPMEI1 and AdPMEI (bottom). Residue of the kiwi inhibitor involved in H-bonds (red), Van der Waals contacts (green) and water-mediated H bonds (yellow) with tomato PME1 and conserved in VvPMEI1 are shown. The secondary structure elements, as elucidated in AdPMEI crystal structure, are boxed in black on the sequence.

Surface plasmon resonance interaction analysis was employed to study the binding between VvPMEI1 and purified tomato PME1. Real-time interaction was measured by injecting various concentrations of VvPMEI1 over a sensor chip with immobilized PME1, and recording the changes in the resonance signal as a function of time. No binding with PME1 could be detected when VvPMEI1 was immobilized on a sensor chip by covalent coupling, possibly because the covalent immobilization of the inhibitor to the sensor chip carboxymethyldextran matrix prevents its interaction with the enzyme (not shown). VvPMEI1 strongly interacts with the enzyme at pH 5.5 (K_D_ 4.77 nM), as shown by the K_off_ value of 3.17x10^-3^ s^-1^ indicating the low dissociation rate of the complex (Fig 5A). The bound VvPMEI1 could be efficiently removed by injections of HBS pH 8.5. At pH 7.5 the equilibrium dissociation constant for the PMEI-PME binding was markedly increased (K_D_ 35nM), mainly due to a faster dissociation with a K_off_ value of 26 x10^-3^ s^-1^ (Fig 5C). Fig 5B and 5D also show that the equilibrium of the complex is reached at lower concentration at pH 5.5 with respect pH 7.5 indicating that the stability of the VvPMEI1-PME1 complex was highly affected by pH. The effect of pH on the kinetics parameters of the interaction was similar to the previously studied complex between AdPMEI and tomato PME1, showing the higher affinity between the two proteins at acidic pHs [48,67]. The elucidation of three-dimensional structure of the complex demonstrated that kiwi AdPMEI binds the tomato PME1 in proximity of the active site cleft [13]. We have built a homology model of VvPMEI1 based on the templates with the highest sequence identity using the Swiss Model algorithm for sequence alignment with statistically significant matches to known 3D structures. Different PMEI/invertase inhibitors were tested as templates, and the different homology models were analyzed in terms of QMEAN (Qualitative Model Energy ANalysis). Only the AdPMEI template produced a model with a satisfactory QMEAN, while all other templates did not reach the threshold value. VvPMEI1 and AdPMEI structures are almost completely superimposable (Fig 6A). There is a clear correlation between sequence and interaction conservation, and the vast majority of interacting pairs above a minimum of about 25% of identity between domains in different chains interacts in the same way [68]. In the model, there is a strong homology in the interface where the majority of contacts between the inhibitor and the enzyme were found in AdPMEI-PME1 complex [13]. The residues of AdPMEI considered crucial for the interaction with PME1 are conserved in VvPMEI1 with the exception of Lys 11, Arg 13 and Asp 140 of AdPMEI (Fig 6B). Interestingly, Asn 101, Asp 109, Thr113 located on α3 helix of AdPMEI, which form intermolecular H-bonds with tomato PME1 [13] are also conserved in VvPMEI1. Moreover, the charged residues that are responsible for the pH dependence of the AdPMEI inhibitory activity and of the AdPMEI-PME1 interaction are conserved in VvPMEI1. The kinetic behavior observed by SPR studies shows that the interaction is modulated by pH changes. Previous observations, based on the AdPMEI-PME1 crystallographic structure, indicated that Thr 113 on the inhibitor may be crucial in determining the strength of PMEI-PME interaction in the pH range 5.0–7.0 [13]. The prediction of the pKA of ionizable residues at different pH values, obtained using the homology model, suggests that also in VvPMEI1 the conserved Thr 118 could be crucial in determining the pH dependence. We can therefore hypothesize that VvPMEI1 binds tomato PME1 with the same surface of interaction of AdPMEI.

### *VvPMEI1* expression in different *V*. *vinifera* tissues

**Fig 7:**
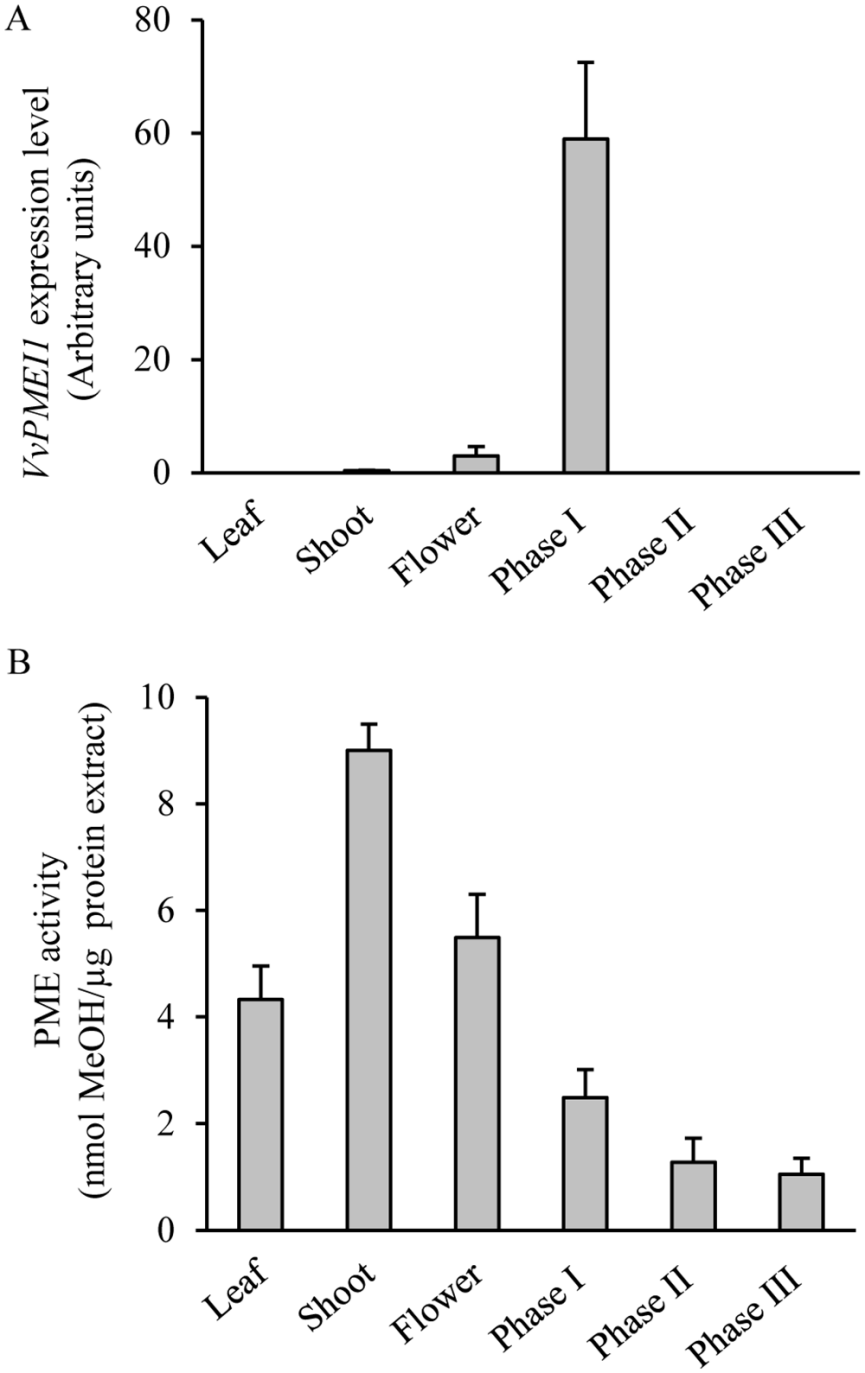
Analysis of VvPMEI1 expression and PME activity in grapevine tissues and during grape berry development. (A) Expression analysis of *VvPMEI1* in various grapevine organs by real-time PCR. The relative level of gene expression was normalized with respect to EF1 mRNA; (B) PME activity. Bars represent the average ± SD (n = 3).

The expression of *VvPMEI1* was assessed in different grapevine tissues and during grape berry development by quantitative real-time PCR. A tissue specific expression of the inhibitor was observed. In particular, the higher *VvPMEI1* expression was observed during berry development, a lower level was detected in flowers while no expression was revealed in leaves and shoots (Fig 7A). The growth of grape berry can be divided into three phases [26,69]. Phase I is a green color stage characterized by a rapid berry growth both through cell division and expansion; phase II, characterized by slow growth, includes the veraison stage at which berry color changes and the softening begins; phase III is the ripening and maturation period [69]. VvPMEI1 is mainly expressed in phase I, (about 20 days after flowering) while no accumulation of *VvPMEI1* transcripts was detected at the later developmental phases II and phase III (respectively, 50 and 100 days after flowering). Consistently, in previous transcriptome analysis the expression of VvPMEI1 was detected at stage I of berry development [70,71]. The PME activity was quantified in the different grapevine tissues. The highest pectin methylesterase activity was detected in shoot, while a lower level was detected in leaves and flowers. During berry development, the level of PME activity is almost comparable with a slight high level in phase I (Fig 7B), as also previously reported[28]. Interestingly, the *VvPMEI1* expression negatively correlates with PME activity in the passage from flowers to phase I of grape berry development. These results, together with our previous observations, suggest that VvPMEI1 can play a role in modulating the PME activity and degree of pectin methylesterification in flower and young berries.

With the aim to identify possible PME isoforms target of VvPMEI1, proteins extracted from flowers and from phases I and II of grape berry development were analyzed by LC-MS/MS (S1 Table). A pectin methylesterase XP_002271665.2 (VIT_07s0005g00730; F6HZ64) isoform was detected in phase I. PME isoform has a very high sequence identity (of about out 75%) with kiwi fruit PME (P87076;[64]) and tomato fruit PME1 (of about 50%) both inhibited by AdPMEI [48]. Interestingly, by aligning the amino acid sequence of the grapevine PME with those of tomato and kiwi PMEs, it becomes evident that all the tomato PME1 residues previously shown to be involved in contacts with the kiwi inhibitor [13] are quite all conserved in the grapevine and kiwi PME isoforms (S4 Fig). VvPMEI1, exogenously added to the proteins extracted from grape berry at phase I, was able to completely inhibit the PME activity supporting its potential implication in the post-transcriptional modulation of the enzymatic activity at the early stages of berry development (S5 Fig). The co-expression of specific PMEIs and PME isoforms during physiological processes indicates possible partners of *in vivo* interaction [3]. Noteworthy, by using the ViTis Co-expression database (http://vtcdb.adelaide.edu.au/home.aspx) VvPMEI1 and the grapevine PME VIT_07s0005g00730 were found to be co-expressed at stage I of berry development [71]. The analysis of expression of VIT_07s0005g00730 transcript focused in flower and during grape berry development was assessed by quantitative real-time PCR. As VvPMEI1 the higher expression of the enzyme was observed at phase I of berry development while lower levels were detected in flowers and in the other stages of berry development (S6 Fig). The observed pattern of expression of the two proteins support the conclusion that the identified grapevine PME could be targeted by VvPMEI1 at early stages of berry development. Our results also reveal the expression of a putative pectin methylesterase inhibitor (CBI24573.3) in flower and of another putative PMEI (CBI24569.3) at phase II of berry growth (S1 Table). Overall, these results suggest that the post-transcriptional regulation of PME activity by PMEIs is involved in the control of pectin methylesterase activity during grape berry development.

The implication of specific PMEI isoforms in the modulation of PME activity at precocious stages of fruit development has also previously reported in tomato, kiwi and banana fruits [18,20,25,29,72]. A control of PME activity by VvPMEI1 at early phases of grape berry development could be required for a rapid cell growth and enlargement and to maintain pulp firmness [26,28]. VvPMEI1 could also be implicated in preventing precocious grape berry softening related to pectin degradation. VvPMEI1 could be involved in pollen tube growth, as previously reported for other PMEIs [50]. Unripe fruits are less susceptible to fungal and bacterial pathogens [33,73]. The expression of VvPMEI1 could contribute in fruit set and young berries to maintain high the pectin methylesterification to hamper the action of microbial pectinases during pathogen infection.

## Supporting Information

S1 Fig Nucleotide and deduced amino acid sequences of the VvPMEI1 gene. Signal peptide is indicated in grey and the stop codon is marked with an asterisk.
(PDF)

S2 Fig Motif analysis of VvPMEI1 5′ flanking region. Yellow indicated AGAAA motif, red indicated CAAT box, green indicated TATA box, blue indicate hormone responsive element and grey indicated defence responsive motifs.
(PDF)

S3 Fig Protein coverage for VvPMEI1 of peptides obtained after trypsin digestion. The peptides identified are reported in green (High confidence) and red (Low confidence) on the coverage map and in bold in the amino acid sequence.
(PDF)

S4 Fig Clustal Omega (http://www.ebi.ac.uk/Tools/msa/clustalo/) multiple sequence alignment between the mature portions of PME F6HZ64 from grapevine, PME1 from tomato and kiwi fruit PME. An * (asterisk) indicates positions which have a single, fully conserved residue. A: (colon) indicates conservation between groups of strongly similar properties. A. (period) indicates conservation between groups of weakly similar properties. In green PME1 residues involved in contacts with AdPMEI; in grey conserved contacting residues.
(PDF)

S5 Fig Inhibitory effect of VvPMEI1 on PME activity at phase I of berry development. PME activity in crude extract of grape berry at phase I of development alone or in presence of VvPMEI1 at the indicated amounts is shown. Bars represent the average ± SD (n = 3).
(PDF)

S6 Fig Analysis of the expression of grapevine PME VIT_07s0005g00730 in flowers and during grape berry development. The expression analysis of VIT_07s0005g00730 in flowers and berries at different developmental stages was performed by real-time PCR. The relative level of gene expression was normalized with respect to EF1 mRNA. Bars represent the average ± SD (n = 3).
(PDF)

S1 Table Proteins identified from grapevine flowers and grape berry at phase I and II of development. Protein identifications were made by MaxQuant against Viridiplantae proteins downloaded from NCBI.
(XLSX)
