# Supplementary material for: The Grapevine VvPMEI1 Gene Encodes a Novel Functional Pectin Methylesterase Inhibitor Associated to Grape Berry Development
Source: PLoS One. 2015 Jul 23;10(7):e0133810. doi: 10.1371/journal.pone.0133810 (PMC4512722; doi:10.1371/journal.pone.0133810)
Supplement: S1 Fig — Signal peptide is indicated in grey and the stop codon is marked with an asterisk. (PDF) [file pone.0133810.s001.pdf]

```
1      ATGGCGTGTCCTGGCATCCACATATCCTCCATGCTAATGGTGTTTTTGTTGTTGATTAAT
1       M  A  C  P  G  I  H  I  S  S  M  L  M  V  F  L  L  L  I  N

61     GGCTCCTTCAGCAGGCCAAGCGTGAAGATCGCAAACAATGAGTTGACTGAGATTTGTTCC
21      G  S  F  S  R  P  S  V  K  I  A  N  N  E  L  T  E  I  C  S

121    ACAACTCAAGATCCTTCGTTTTGTGTTCAAGCTTTGAAGTCTGATCCTCGCACTGCCAAC
41      T  T  Q  D  P  S  F  C  V  Q  A  L  K  S  D  P  R  T  A  N

181    GCTGACCTCAAAGGCCTGGCCCAAATCTCTATCGACTTGGCCAAGGCCAGTGCTACAAAA
61      A  D  L  K  G  L  A  Q  I  S  I  D  L  A  K  A  S  A  T  K

241    ACCACTACTTTGATCACCTCTCTGGTGGAAAAAGCCAATGATCCTAAGCTTAAAGGACGC
81      T  T  T  L  I  T  S  L  V  E  K  A  N  D  P  K  L  K  G  R

301    TATGAAACGTGTGCAGAAAACTATGATGATTCCATAAGTTCATTGGATGATTGTACTCAA
101     Y  E  T  C  A  E  N  Y  D  D  S  I  S  S  L  D  D  C  T  Q

361    TCTGTGTCTTCTAGAGACTATGTTAGTTTGAATTTTCAAGCGTCTGCTGCCATGGATGGA
121     S  V  S  S  R  D  Y  V  S  L  N  F  Q  A  S  A  A  M  D  G

421    CCTGTGACATGCTTAGATAGCTTTGAAGGACCACCAAAGGACCCATCTGAACTGCCTACC
141     P  V  T  C  L  D  S  F  E  G  P  P  K  D  P  S  E  L  P  T

481    AAGTCTGAAGATTTAATACATCTTTGTAGCATCATTTTGGCTATCTCTAAGCGTTTGATA
161     K  S  E  D  L  I  H  L  C  S  I  I  L  A  I  S  K  R  L  I

541    GGATAA
181     G  *
```
