# Supplementary material for: The Grapevine VvPMEI1 Gene Encodes a Novel Functional Pectin Methylesterase Inhibitor Associated to Grape Berry Development
Source: PLoS One. 2015 Jul 23;10(7):e0133810. doi: 10.1371/journal.pone.0133810 (PMC4512722; doi:10.1371/journal.pone.0133810)
Supplement: S2 Fig — Yellow indicated AGAAA motif, red indicated CAAT box, green indicated TATA box, blue indicate hormone responsive element and grey indicated defence responsive motifs. (PDF) [file pone.0133810.s002.pdf]

VvPMEI 5'UTR

ATATTTGATTTTCTTTCATATGTTTTATAATAAAATYAAACATGATGGGAATAAAAAATAATCTAAATCAAAA
GAAATAGAGCCCACCAATAAATATAATTTTGAAAATATTAATAGCACTTCCAAAACAATGTTAAATATTAAA
ATTATTTTTTTAAATGATAAGAAATAGTGATTCTTAAACTATATTTAATGCATTCTTCTTTTCTTTTCTTTTCTT
TTAAGAATTATCTTTGTTTTAATGGCACATTTACAAAAATATTATTTTGATTCGTATCAGAATATTAACTGATA
TTCTATAGAATGTTTTTTATATAAATACTTTTATCAAATAAATAAATTCTACATATTTCATTTAATTGTCATTTT
TAAAATTAGATATAACATATAAATATTAATTTTAAATTTTTAATTACACCTATTCAATTTTTTTTAACTCATGTT
TCAAGTTCTTTTCATTAAAAAATACAAAAATTCATAAATAAATAAATAAATAATTCATGGTCCTTTTGGTAAA
AAAATTTGGTTGAAAACAATTCATATTGATTAGTTTTCTATGCATTGTACAAAATATTCTTTTAATTGAATTA
ATTTTTTTTATATGTTTCATGTTCATTTTATTAAAAAAAAAATTAATTATGTTCATCTAGGTAAAAATACAAAT
AAAGAAATTAAAAAGAATAAAAAAAAATTCTGTTTCAATTGAAAATATATTCATTAATGGGCTTTTTTTTTA
GATAATTGAATAAATTGAAATTCTTTAAAAATATTTGTGGTCTTTTAAATATTTAAATGATTATCAATTAAAA
CGAGTAATGTATAATTTCTTTGCAGCACAATTATTTTTATCCTAAAAGTTAGTTTTTTATATTTGTCAATAAAAT
AAAATATAATTATTTACAAAATATTTATTATAAATTTGGAAAATTTTCAAGGGGACATAACCTTGAAAGTTTC
AAAAAAAGTTTCTAAATAATTATTTTTACCCTAGAACATTTTAGAAAATATGTTCATGCATGGTTAGAGAAA
ATTATTTACAAAAAGGGAATGGAGATGCATACCATGTTAATGAAAAAAAAWAATAATAATAATAAACTAG
GTAGGGGCCAAATTAATGAAATAGGAAAGGTTCAATCACATACCAAACTTAGAAAAAAATAAAATAAAATC
TAAATTATATTTTTAGAAATGTAAGATTTTTTGCCAATAATAACGTAATATCTCATCATATATGCAGTATTAG
GCACAAATTAACATATATAGAAGATGATATTTGGGTTATATAAATATAAGGAATGAGTTGATGTCTTATTTC
AAAGAAATTCAAACAAAAGAAGGAGAAAA
