# Supplementary material for: The Grapevine VvPMEI1 Gene Encodes a Novel Functional Pectin Methylesterase Inhibitor Associated to Grape Berry Development
Source: PLoS One. 2015 Jul 23;10(7):e0133810. doi: 10.1371/journal.pone.0133810 (PMC4512722; doi:10.1371/journal.pone.0133810)
Supplement: S3 Fig — The peptides identified are reported in green (High confidence) and red (Low confidence) on the coverage map and in bold in the amino acid sequence. (PDF) [file pone.0133810.s003.pdf]

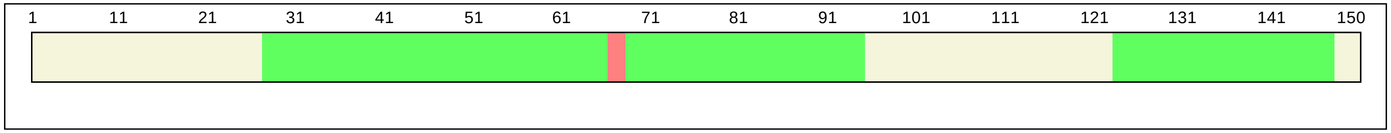

PSVKIANNE LTEICSTTQD PSFCVQALKS DPR**TANADLK**
GLAQISIDLA KASATKTTTL ITSLVEKAND PKLKGRYETC
AENYDDSISS LDDCTQSVSS RDYVSLNFQA SAAMDGPVTC
LDSFEGPPKD PSELPTKSED LIHLCSIILA ISKRLIG
