# Supplementary material for: The Grapevine VvPMEI1 Gene Encodes a Novel Functional Pectin Methylesterase Inhibitor Associated to Grape Berry Development
Source: PLoS One. 2015 Jul 23;10(7):e0133810. doi: 10.1371/journal.pone.0133810 (PMC4512722; doi:10.1371/journal.pone.0133810)
Supplement: S4 Fig — An * (asterisk) indicates positions which have a single, fully conserved residue. A: (colon) indicates conservation between groups of strongly similar properties. A. (period) indicates conservation between groups of weakly similar properties. In green PME1 residues involved in contacts with AdPMEI; in grey conserved contacting residues. (PDF) [file pone.0133810.s004.pdf]

```
CLUSTAL O(1.2.1) multiple sequence alignment

Grapevine   TR|F6HZ64|F6HZ64_VITVI    IHYNLTVAKDGSGDFTTIGEAIAAAPNSSTTRFVIHIKAGAYFEY 288
Tomato      SP|P14280|PME1_SOLLC      IIANAVVAQDGTGDYQTLAEAVAAAPDKSKTRYVIYVKRGTYKEN 274
Kiwi        SP|P85076|PME_ACTDE       IVPDVVVAKDGSGNFTTVGAAVAAAKDSSTARFVIYIKEGAYFEY 50
                                      *  : .**:**:*:: *:. *:*** :.*.:*:**::* *:* *

TR|F6HZ64|F6HZ64_VITVI LDIARSKTMLMLVGDGLENTYIKGNRSVGGGWTTFQSGTVAVVANNFIAKGISFENYAGP 348
SP|P14280|PME1_SOLLC   VEVASNKMNLMIVGDGMYATTITGSLNVVDGSTTFRSATLAAVGQGFILQDICIQNTAGP 334
SP|P85076|PME_ACTDE    VDVDKKKTNLMFIGDGIGKTWIKGNRSVVDGWTTFRSSTVAVVGTGFIARGISFENYAGP 110
                       :::  .*  **::***:  * *.*. .*  * ***:*.*:*.*.  ** : *.::* ***

TR|F6HZ64|F6HZ64_VITVI SNHQAVALRSGADLSVFYLCRFIGYQDTLYVHSLRQFYRECDVYGTIDFIFGNAAVVLQN 408
SP|P14280|PME1_SOLLC   AKDQAVALRVGADMSVINRCRIDAYQDTLYAHSQRQFYRDSYVTGTVDFIFGNAAVVFQK 394
SP|P85076|PME_ACTDE    SKHQAVALRSGADFSAFYQCSFVGYQDTLYVHSLRQFYSECDVYGTIDFIFGNAAAVLQK 170
                       ::.****** ***:*.:  * : .******.** **** :. * **:********.*:*:

TR|F6HZ64|F6HZ64_VITVI CNLYARRPNANQKNVFTAQGRDDPNENTGISIQNCKVAAAADLIPVLSSFKSYLGRPWKE 468
SP|P14280|PME1_SOLLC   CQLVARKPGKYQQNMVTAQGRTDPNQATGTSIQFCNIIASSDLEPVLKEFPTYLGRPWKE 454
SP|P85076|PME_ACTDE    CNLYARKPNENQKNIFTAQGRDDPNQNTGISILNCKVAAAADLIPVLSSFKTYLGRPWKE 230
                       *:* **:*   *:*:.***** ***: ** **  *:: *::** ***..* :********

TR|F6HZ64|F6HZ64_VITVI YSRTVYMQSNIGNLIDPAGWLEWDGDFALSTLYYGEYKNRGPGSNTSGRVTWPGYRVINS 528
SP|P14280|PME1_SOLLC   YSRTVVMESYLGGLINPAGWAEWDGDFALKTLYYGEFMNNGPGAGTSKRVKWPGYHVITD 514
SP|P85076|PME_ACTDE    YSRTVFLLSQMESLIDPAGWLEWSGDFALTTLYYREYKNTGPGSNTTARVTWPGYAVTTN 290
                       ***** : * :  **:**** **.*****.**** *: * ***: *: **.**** * ..

TR|F6HZ64|F6HZ64_VITVI SSVASQFTVGAFIQGDEWLPATGIPYYSNLTIV 561
SP|P14280|PME1_SOLLC   PAKAMPFTVAKLIQGGSWLRSTGVAYVDGLYD- 546
SP|P85076|PME_ACTDE    ETEVIQFTVGNFIQGSQWLTSYNIPVYLNLT-- 321
                        : .  ***. :*** .** :  :     *
```
