# Supplementary figures and images for: The Grapevine VvPMEI1 Gene Encodes a Novel Functional Pectin Methylesterase Inhibitor Associated to Grape Berry Development
Source: PLoS One. 2015 Jul 23;10(7):e0133810. doi: 10.1371/journal.pone.0133810 (PMC4512722; doi:10.1371/journal.pone.0133810)

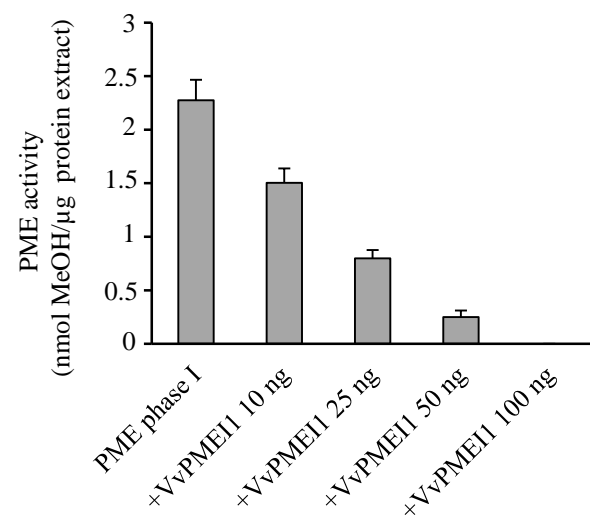

Supplement: S5 Fig — PME activity in crude extract of grape berry at phase I of development alone or in presence of VvPMEI1 at the indicated amounts is shown. Bars represent the average ± SD (n = 3). (PDF) [file pone.0133810.s005.pdf]

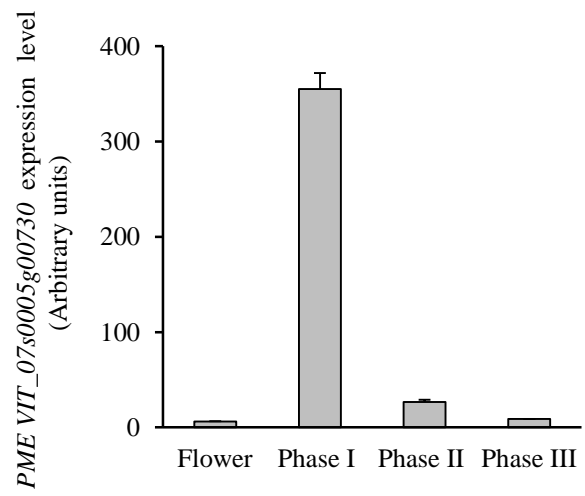

Supplement: S6 Fig — The expression analysis of VIT_07s0005g00730 in flowers and berries at different developmental stages was performed by real-time PCR. The relative level of gene expression was normalized with respect to EF1 mRNA. Bars represent the average ± SD (n = 3). (PDF) [file pone.0133810.s006.pdf]
